# Numerical and Experimental Analysis of Mechanical Properties in Hybrid Epoxy–Basalt Composites Partially Reinforced with Cellulosic Fillers

**DOI:** 10.3390/ma16144898

**Published:** 2023-07-08

**Authors:** Vijay Chandan, Rajesh Kumar Mishra, Viktor Kolář, Petr Jirků, Miroslav Müller, Hafsa Jamshaid

**Affiliations:** 1 Department of Material Science and Manufacturing Technology, Faculty of Engineering, Czech University of Life Sciences Prague, Kamycka 129, Suchdol, 165 00 Prague, Czech Republic; vijay@tf.czu.cz (V.C.); vkolar@tf.czu.cz (V.K.); jirkup@tf.czu.cz (P.J.); muller@tf.czu.cz (M.M.); 2 Faculty of Textile Engineering, National Textile University, Faisalabad 37610, Pakistan; hafsa@ntu.edu.pk

**Keywords:** hybrid composites, cellulosic/bio-fillers, basalt woven fabrics, mechanical properties, modulus

## Abstract

The current work is focused on numerical and experimental studies of woven fabric composites modified by hybridisation with biological (cellulosic) filler materials. The mechanical performance of the composites is characterized under tensile, bending and impact loads and the effect of hybridisation is observed with respect to pure and nonhybrid composites. Numerical models are developed using computational tools to predict mechanical performance under tensile loading. The computational prediction results are compared and validated with relevant experimental results. This research is aimed at understanding the mechanical performance of basalt–epoxy composites partially reinforced with micro-/nano-sized bio-fillers from cellulose and intended for various application areas. Different weave structures, e.g., plain, twill, matt, etc., were investigated with respect to the mechanical properties of the hybrid composites. The effects of hybridizing with cellulose particles and different weave patterns of the basalt fabric are studied. In general, the use of high-strength fibres such as basalt along with cellulosic fillers representing up to 3% of the total weight improves the mechanical performance of the hybrid structures. The thermomechanical performance of the hybrid composites improved significantly by using basalt fabric as well as by addition of 3% weight of cellulosic fillers. Results reveal the advantages of hybridisation and the inclusion of natural cellulosic fillers in the hybrid composite structures. The material developed is suitable for high-end applications in components for construction that demand advanced mechanical and thermomechanical performance. Furthermore, the inclusion of biodegradable fillers fulfills the objectives of sustainable and ecological construction materials.

## 1. Introduction

Recently, polymer-based composite materials have developed a wider significance in many areas of application, and research in this area is gaining more and more importance [1,2]. However, while experimental investigations have proved fruitful, it is not simple to accurately predict the performance of the final composite product [3,4,5].

Woven fabrics are used as reinforcement in composites because of their symmetry and balanced properties in the fabric plane as well as their ease of handling during the fabrication of the composites. However, having a two-dimensional structure, these fabrics show poor mechanical properties in the thickness direction and their reinforcement in composites results in relatively lower impact properties [6,7]. In order to develop composites with better impact performance, significant work has been done on increasing the thickness of fabrics by engineering the woven structures or by simply layering the 2D fabrics. However, despite increased thickness after layering, previous studies found no clear trend in the improvement of impact properties because of delamination of individual layers [8,9,10,11]. Recently, an approach centred on adding micro/nanofillers to composites is being practiced to improve the delamination resistance of composites, albeit at an increased cost due to the additional filler material. Therefore, in the last few years, hybrid composite structures have created a lot of interest in technical applications where properties in the thickness direction are extremely important [12,13,14,15].

Basalt fibres have been used in composite materials due to their advanced mechanical, thermal and thermomechanical performance [16,17,18,19]. Since they are derived from minerals, they are considered an eco-friendly alternative to the high-performance synthetic fibres used in the composites industry. However, to date, there has been little in-depth study of basalt-based composite materials in polymer matrices [20].

Since fibrous/textile-based reinforcement does not provide enough orientation of fibres in the thickness direction, there is inferior mechanical performance in the bulk direction. By plying several layers of fabrics, one cannot completely eliminate the risk of delamination under severe mechanical loading conditions. Therefore, an alternative approach is to combine or hybridize fibrous reinforcement with filler materials. Using this approach, the mechanical, thermal and thermomechanical performance can be both controlled and improved in the bulk/thickness direction [21,22,23,24]. Hybrid composites in several instances have offered improved impact resistance under low-velocity loading [25,26]. They can be engineered to provide sufficient mechanical performance in the preferential direction.

The cost factor associated with high-performance fibres can be offset by hybridisation with low-cost biological materials, e.g., cellulosic waste from plant products [27]. The hybrid effect can complement the performance of the fibres and the fillers [28,29]. Such practice is common in metallurgy but to date, little research has been reported in the area of hybridizing high-performance fibres and cellulosic fillers in polymer composites [30,31,32,33].

Epoxy resins are popularly used in composite applications as they do not give off reaction products while curing and have low cure shrinkage [34]. They show a number of outstanding characteristics such as high strength and stiffness and good chemical and electrical resistance in addition to very good adhesion to fibres and inorganic substrates [35,36,37]. However, because of their brittle nature, they have poor crack resistance and low impact strength. Therefore, in the last few decades, the toughening of epoxies has received much attention [38]. There are several methods to overcome the brittleness of epoxy resins, such as the use of chemical modifiers [39], reducing the cross-linking density of the epoxy network, or modifying the epoxy resin with secondary components, e.g., bio-fillers [40,41,42]. A simple and effective way to improve fracture energy without loss of strength and elastic modulus is via incorporating micro-/nano-sized fillers such as cellulosic fillers. Thus, in addition to delamination resistance, the role of cellulose could also be studied in terms of the improvement of the toughness of epoxy [43].

The interfacial bond between textile fabric reinforcement and the composite matrix is highly heterogeneous due to the orientation of the fibres/yarns in the x and y directions. Therefore, it is essential to characterize the bond behaviour in order to understand the macroscale behaviour of a fibre-reinforced composite material [44]. Several researchers have reported the advantages of textile fibres and woven fabrics in improving the mechanical performance of composite materials. However, the influence of hybridizing woven structures with micro/nanofillers has not been extensively studied experimentally [45,46,47].

The primary objective of this work is to study the role of various basalt fabric constructions engineered to improve the properties of composites in the thickness direction in the form of composites hybridized with micro/nanocellulosic fillers. The second objective is the utilisation of bio-origin, low-cost fillers such as cellulose for the improvement of the tensile and bending, as well as impact, performance of hybrid composites. The investigations are focused on the reinforcement of basalt woven fabrics with plain, twill and matt constructions. Numerical modelling is done to predict the tensile performance of the developed composites and the predicted results are compared with experimental data. The combined effect of a varying weave structure and an optimized concentration of cellulosic fillers are investigated to develop hybrid composite samples with enhanced mechanical and thermomechanical performance.

## 2. Materials and Methods

### 2.1. Materials

Basalt is a fibre originating from rocks and has excellent mechanical and thermal performance. The basalt yarns used in this research were sourced from Kamenny Vek (KV) (Shelby, NC, USA). The details of fibres and yarns are given in Table 1.

Green epoxy resin CHS-G530 (new commercial name EnviPOXY^®^530) was purchased from the company Spolchemie in Ústí nad Labem, Czech Republic. Chemically, it contains 4,4′-Isopropylidenediphenol, oligomeric reaction products with 1-chloro-2-3-epoxypropane. It is also called “green” due to the synthesis of 1-chloro-2-3-epoxypropane (epichlorohydrine) from glycerine which originates from waste generated during biodiesel production. The properties of epoxy resin are given in Table 2.

### 2.2. Methods

**Figure 1:**
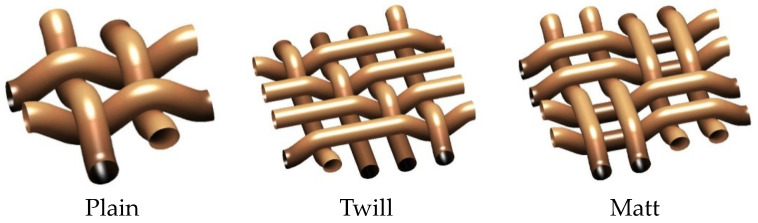
Fabric structures developed.

The basalt fabric samples were made on the CCI sample loom (CCI, Taiwan) with the same thread density for all fabric structures. A total of 12 threads/cm in warp and 8 threads/cm in weft were maintained. The weave structures are shown in Figure 1.

### 2.3. Prediction of Tensile Properties Using Numerical/Computational Models

The software package WiseTex (Leuven, Belgium) was used to predict the mechanical performance of composites with various basalt fabric structures. It is a computational tool for modelling the internal geometry and mechanical properties of composite structures as well. The internal geometry of composites is based on the hierarchical principle of and geometries of the reinforcement. The simulation algorithm uses the minimum energy principle, calculating the equilibrium of yarn interactions in the composites. The models cover a wide range of structures, which are either relaxed or after compression, shear or tensile deformation. The models were developed for the internal geometry of plain, twill and matt weaves [48,49,50,51], based on a minimum number of binding elements (weave style, inter-yarn distance) and yarn mechanical properties. It is a mechanical model, as it applies a minimization algorithm to yarn deformation energy in order to define the internal geometry of any weave and then predict the mechanical performance of composites.

### 2.4. Grinding of Microcellulose by Ball Milling

Cellulose micro fillers were obtained from Havel composites, (Svésedlice, Czech Republic). The cellulose micro-particles were subjected to mechanical grinding in a ball milling device, Fritsch Pulverisette 7 (Kyjov, Czech Republic). The material was ground for up to 5 h in order to obtain the nanosized fillers. The nanofillers were dispersed in deionised water. Bandelin ultrasonic probe (Merck Life Science spol. s r.o., Prague, Czech Republic) was used for mixing the dispersion before characterisation of particle size. The cellulosic nanofillers were dried in an oven at 120 °C for 6 h.

### 2.5. Fabrication of Hybrid Composite Samples by Hand Layup Process

The composite samples were developed by hand layup method maintaining a 50:50 weight ratio of fabric to the matrix. While using cellulosic fillers, the weight fraction of epoxy resin was reduced proportionally but the fabric weight fraction was kept at 50%. Weighed amounts of dried cellulose fillers (1, 3, 5 and 10 wt%) were mixed with epoxy resin at room temperature in order to prepare a homogeneous mixture. The mixture was subjected to ultrasonic stirring for 30 minutes using a Bandelin Sonoplus (Merck Life Science spol. s. r.o., Prague, Czech Republic) device.

**Figure 2:**
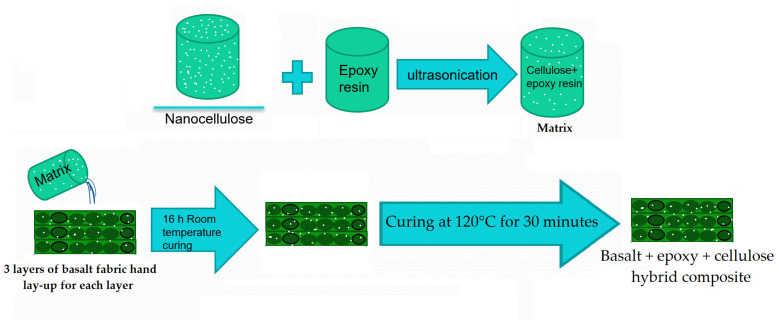
Schematic of hybrid composite sample preparation.

The matrix was prepared with resin, hardener, and cellulosic fillers. A mixture of epoxy and hardener (CHS-Hardener P11, Spolchemie in Ústí nad Labem, Czech Republic) was prepared as per manufacturer guidelines with a ratio of 100:32 and stirred well for uniform mixing. The viscosity of (resin + hardener) was found to be 10 Poise at 25 °C, the viscosity for (resin + hardener + microcellulose) was 11 Poise and that for (resin + hardener + nanocellulose) was 9 Poise at 25 °C. The decrease in viscosity due to the addition of nanoparticles does not follow Einstein–Stokes equation and is well documented in the literature [50,51]. In order to develop the composite samples, a mold of 20 cm × 20 cm (length × width) and 3 mm thickness was prepared. Layers of the fabrics were placed within the boundaries of the mold. In order to ensure uniform resin application, the spreading was performed layer by layer so that the averaging effect minimizes the variation. No visible voids were found in the prepared samples. The matrix was dispensed over the fabric layers in the mold very carefully to ensure uniform distribution throughout the sample. A Teflon sheet was placed on both sides of the material in order to keep them intact. It was followed by the curing of the samples at room temperature for 16 h. Further, the samples were cured for 30 minutes at 120 °C under 100 bar pressure. After curing under pressure, the thickness was measured for each sample and was found to be within the limits. The schematic of the hybrid composite sample preparation is shown in Figure 2.

### 2.6. Testing of Mechanical Properties

For testing purposes, the samples were cut using high-energy water jet cutting technology (CNC cutting machine AWJ CT 0806, Cutting Trading International, Castelfranco Veneto, Italy). Therefore, no edge errors were detected.

The testing of mechanical properties was carried out on the universal testing machine LABTest 5.50 ST (LABORTECH s.r.o., Opava, Czech Republic), with the measuring unit AST KAF 50 kN (LABORTECH s.r.o., Opava, Czech Republic) and the evaluation software Test & Motion (version 4.5.0.15, LABORTECH s.r.o., Opava, Czech Republic) as per ASTM D3039/D3039M-08 standard [52]. The tensile test was carried out at a speed of 12mm/minute. The specimen size of 100 mm × 10 mm was used for the samples. Each test was repeated 20 times and the average was calculated.

**Figure 3:**
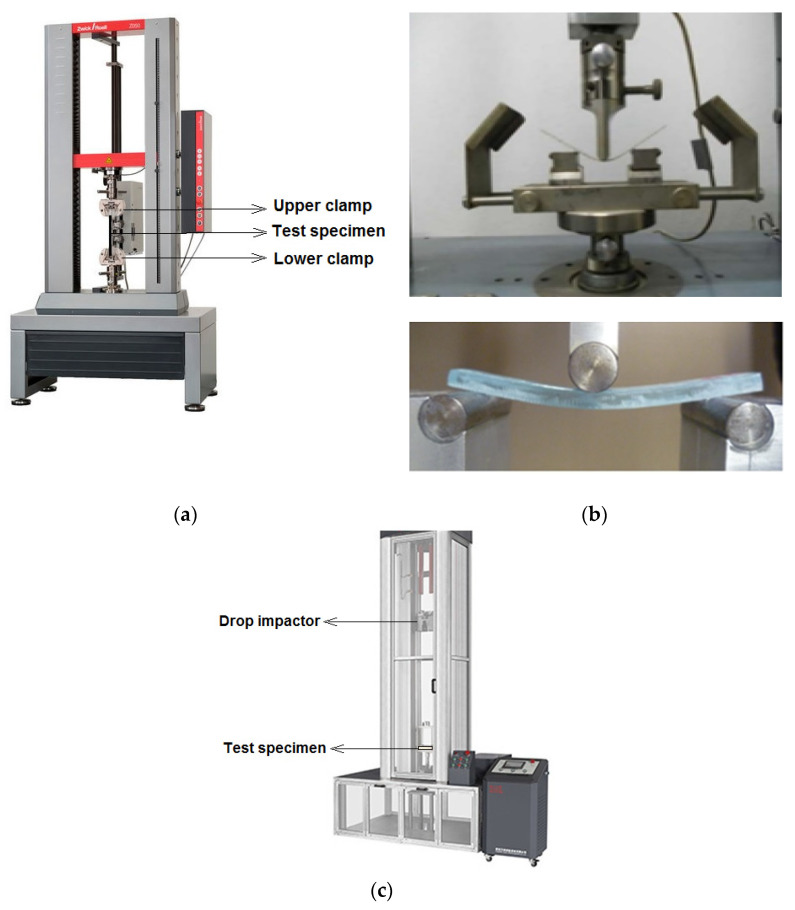
Test setups for (**a**) tensile test, (**b**) 3-point bending test and (**c**) impact test.

Flexural strength was measured on LABTest 5.50 ST (LABORTECH s.r.o., Opava, Czech Republic), using a load-cell of 500 N capacity in the 3-point bending mode as per ASTM D7264/D7264M-07 standard [53]. The sample dimensions were entered into the system and the stress–strain curves were recorded for all samples. The specimen size for this test was kept as 100 mm × 10 mm. Bending test for each type of sample was repeated 20 times. Testomeric tester M350-10CT (LABORTECH s.r.o., Opava, Czech Republic) with ball impactor was used to assess the impact resistance of composite samples following ASTM D7136/D7136M-05 standard [54]. The impactor was allowed to strike the samples at a velocity of 100 m/s using a 100kg load-cell. The specimen size for this test was kept as 100 mm × 100 mm. Each test was repeated 20 times and the average was calculated. The experimental setups for the tensile, the bending and impact test are shown in Figure 3.

### 2.7. Scanning Electron Microscopy (SEM) Analysis

SEM analysis was performed using a TESCAN VEGA 3 XMU (TESCAN ORSAY HOLDING a.s., Brno, Czech Republic) The microscopic samples were coated with gold using Quorum Q150R ES Plus (Quorum Technologies—Judges House, Laughton, UK).

### 2.8. Thermo-Mechanical Analysis (DMA)

The dynamic mechanical analysis of the hybrid composite materials was performed on the DMA DX04T RMI instrument (R.M.I., Pardubice, Czech Republic). The test was performed in three-point bending mode with gauge length and sample width of 30 mm and 10 mm, respectively. The samples were subjected to an oscillating frequency of 1 Hz and 100% oscillating amplitude in the temperature range of 30 °C to 300 °C at a heating rate of 5 °C min^−1^.

## 3. Results and Discussion

### 3.1. Effect of Ball Milling on the Size of Cellulosic Filler

**Figure 4:**
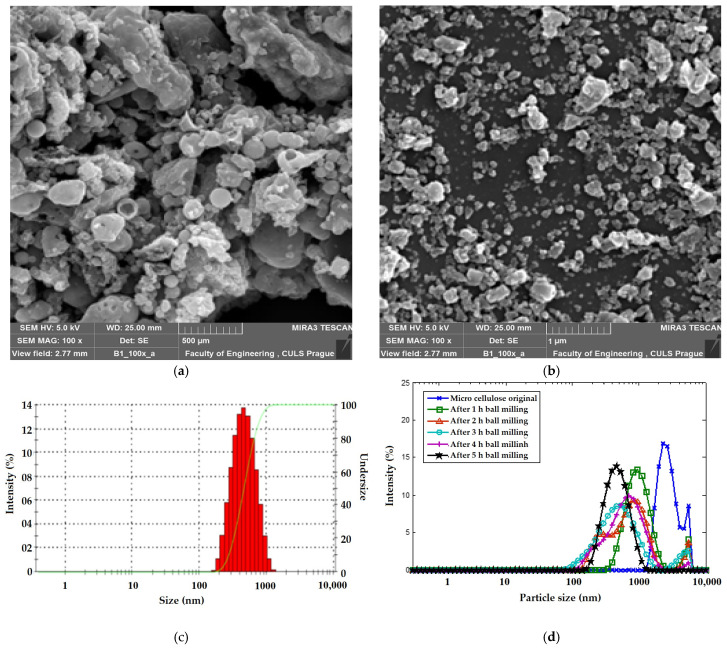
(**a**) SEM of cellulosic micro fillers, (**b**) SEM of cellulosic nanofillers, (**c**) distribution of filler size after milling and (**d**) effect of milling time on filler size.

Figure 4a,b show the SEM images of micro- and nano-sized cellulosic fillers. It is visible that the spherical shape of the original microcellulose is broken and an irregular nanoscale filler with a higher active surface is obtained. It is established by previous research that a non-spherical and irregular surface of the filler offers a much higher specific surface area for better interaction with the surrounding matrix [55,56,57,58,59,60]. Figure 4c,d show the filler size distribution curves of the milled cellulose after time intervals of 1, 2, 3, 4 and 5 hours, respectively. It can be seen that the rate of filler size reduction was highest during the initial one hour of milling, during which the characteristic filler diameter (Z-average) reduced from 3547 nm to 989 nm. However, the filler size was gradually reduced later and reached 450 nm after five hours of wet milling as shown in Figure 4d. When milling was performed for a longer time, filler size distribution changed from multimodal to near unimodal distribution.

### 3.2. Numerical Modelling of Tensile Properties Using Wisetex

Numerical models were adopted for the prediction of tensile properties in composite samples. Woven fabrics are composed of orthogonally interlaced yarn elements, and they can be further subdivided into basic elemental structures which can be numerically modelled to predict the tensile properties. Modelling the different layers of fabric as laminates and computing the equivalent laminate properties results in approximate values for the in-plane properties of the fabrics. Further, the property of the resin was implemented along with the fabric reinforcement for predicting the tensile performance of the composites. The Halpin–Tsai model for the calculation of basic mechanical properties in a composite was adopted. The tensile properties of the reinforcing fabrics and matrix were tested and then the tensile properties of the composite samples were calculated using the rule of mixture and Halpin–Tsai model. The Halpin–Tsai model is widely used to predict the effective tensile strength and modulus for fibre-reinforced composites with perfect fibre alignment as reported by several researchers [61,62,63,64]. The details of derivation for the Halpin–Tsai equations are reported in the literature [65]. The Halpin–Tsai equation has the following form:

(1) $$K_{c}=K_{m}\left[\frac{1+\xi \zeta V_{f}}{1-\eta V_{f}}\right]$$

(2) $$\mathrm{With}\;\eta =\left[\frac{(K_{f}/K_{m})-1}{(K_{f}/K_{m})+\zeta }\right]$$

where *K_c_* represents the effective tensile property of the composite while *K_f_* and *K_m_* are the corresponding fibre and matrix tensile properties, *V_f_* denotes the fibre volume fraction and *ζ* is a geometrical parameter, which represents the reinforcement geometry (plain, twill and matt), packing geometry, and loading conditions. In the present analysis, the geometry is defined by the weave pattern. While predicting the tensile properties of samples filled with cellulosic particles, the property of the cellulose-filled matrix (epoxy resin + cellulosic filler) was considered.

The tensile modulus of a hybrid composite can be calculated by using the rule of the mixture if the corresponding values for the constituent fibre and matrix are known. The tensile modulus of a basalt fabric reinforced composite in a longitudinal (parallel) direction can be calculated based on Equation (3).

(3) $$E_{c}=E_{f}V_{f}+E_{m}(1-V_{f})$$

where *E_c_* represents the elastic modulus of the composite, *E_f_* is the elastic modulus of fibre, *E_m_* is the elastic modulus of the matrix (epoxy resin + cellulosic filler) and $V_{f}$ is the volume fraction of fibre/fabric in the composite.

**Figure 5:**
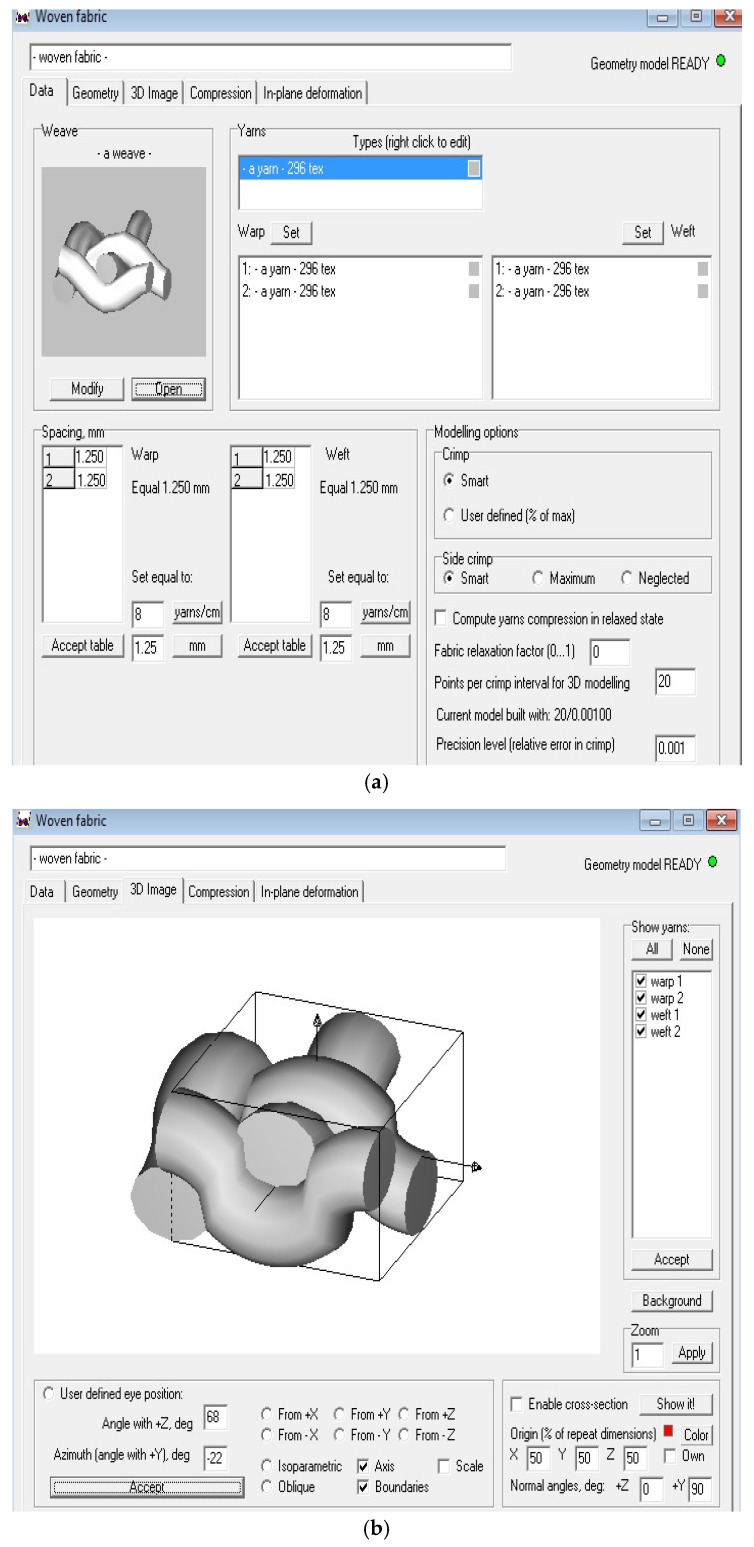

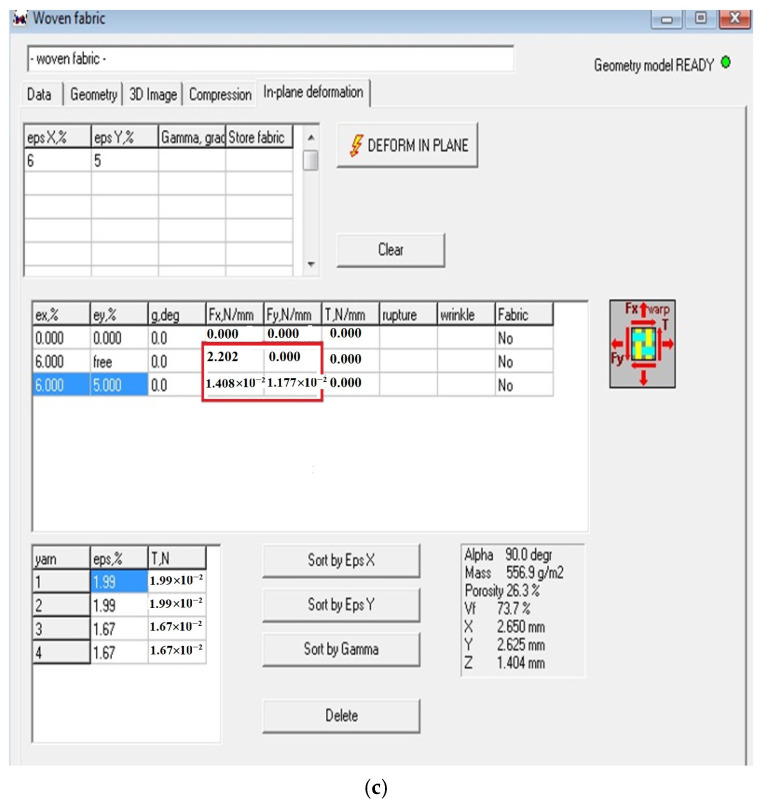
Prediction of tensile properties in composites: (**a**) defining the parameters of fibre and matrix, (**b**) defining the geometrical parameters and (**c**) prediction of tensile properties in composites.

The representative models are shown in Figure 5.

The composite samples were prepared with pure resin as well as with cellulosic fillers. For comparison purposes, both micro- and nanocellulosic fillers were used in 1, 3, 5 and 10% wt in the matrix based on the previous literature [46]. The purpose was to understand the extent of improvement when the resin is reinforced with the cellulosic filler and then the hybrid effect of fabric as well as cellulosic filler in the final composite. The control/reference sample with pure resin was also tested to estimate the % improvement by the hybrid reinforcement. The details of the samples developed are given in Table 3.

**Figure 6:**
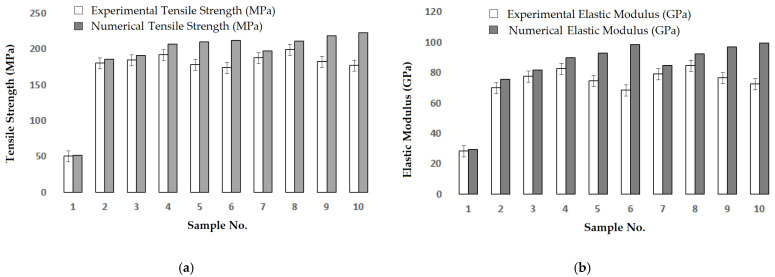
Comparison of experimental vs. numerical tensile properties of composites: (**a**) tensile strength, and (**b**) elastic modulus.

The data obtained from the experimental measurement of 20 samples was statistically analysed and the mean values are presented with standard error. The coefficient of variation was found to be within the 5% limit; therefore, the results and the differences are statistically significant. The tensile strength and modulus of the samples measured experimentally were compared with the numerical models. The results of the tensile test are shown in Figure 6.

The experimental tensile strength and elastic modulus were compared with the results of the numerical analysis, resulting in a high level of agreement between them. The correlation between numerical and experimental results for tensile strength was found to be 75.25% and the correlation of elastic modulus was found to be 77.52%. The important reason for the uncertainty of the results is due to the assumption of boundary conditions and numerical approximation in the models, which do not consider the micro details of the contact between the fibres and the matrix. Moreover, there exist experimental irregularities and dissimilarities which are unavoidable. It was observed that the inclusion of cellulosic micro/nanofillers up to 3% of wt in the matrix improves the tensile strength. However, a further increase in the amount of cellulosic filler deteriorates the experimental mechanical properties which are not accurately predictable by the numerical models. The reason could be due to the irregular dispersion of cellulosic fillers at a higher concentration. Further, it was observed that milled nanofillers of cellulose are more effective in enhancing the mechanical performance of the hybrid composites as compared to unmilled micro-sized cellulosic fillers. This is attributed to the enhanced specific surface area when micro fillers are broken into nanofillers during ball milling [66,67,68,69,70]. Therefore, in further analysis, only milled nanofillers were considered.

### 3.3. SEM Fracture Surfaces

**Figure 7:**
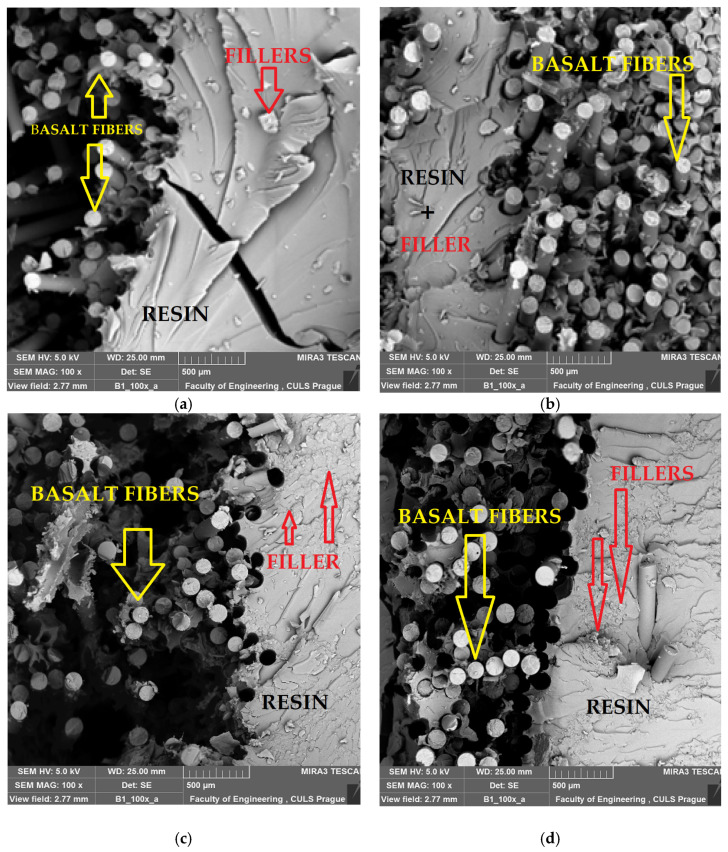
Scanning electron microscope (SEM) images of fractured hybrid composites reinforced with cellulosic fillers, (**a**) basalt + epoxy + 1% nanocellulose, (**b**) basalt + epoxy + 3% nanocellulose, (**c**) basalt + epoxy + 5% nanocellulose and (**d**) basalt + epoxy + 10% nanocellulose.

The SEM fractographs of the hybrid composites partially reinforced with wet-milled cellulosic fillers are depicted in Figure 7. It can be found that the dispersion of cellulosic fillers in the matrix is quite good, and the cellulosic fillers disperse well within the matrix except for a few rich regions generated from nonuniform dispersion of cellulosic fillers at higher loadings, especially beyond 3%.

The basalt fibres, resin and cellulosic fillers are identified and described in the SEM images. It can be observed that, only at 3% filler content, is there adequate bonding between fibres and the matrix, and no visible delamination was found. The fibre matrix interface is optimum at 3% of filler content. The filler and resin separation is visible in the pictures only when there is a lack of supersaturation (1% content) or nonuniform dispersion due to high filler content (5% and 10%). This can be a technological error in the production of samples with higher filler content. Cellulosic nanofillers are rigid with much higher fracture strength as compared to epoxy resin. Therefore, the samples reinforced with 3% fillers did not fracture by the crack growth. A large number of cellulosic fillers were observed on the fractured surface. This indicates that during the process of fracture, the growing crack front has to change its path many times after interaction with cellulosic fillers. This leads to an increase in effective crack length and, hence, higher absorption of energy in the process of fracture [71]. A curvilinear tendency for crack progression was seen for hybrid composites partially reinforced with cellulosic fillers. Thus, the total crack length is highest for the 3% cellulose-filled hybrid composites and is smallest for 1% cellulose-filled composites.

Further, since the addition of 10% cellulosic filler in the matrix was visibly inefficient and ineffective, further analysis of mechanical performance was carried out only for 1%, 3% and 5% nano-cellulosic fillers in the hybrid composites.

### 3.4. Flexural Tests

The flexural stress–strain of composite samples was obtained using a 3-point bending test according to the standard test method ASTM-D7264. The machine speed was maintained at 1 mm/minute and the bending force was exerted on the samples until the peak load reached 40% of the maximum force [72].

The LABTest 5.50 ST (LABORTECH s.r.o., Opava, Czech Republic), a universal testing device was used by changing the clamps. It measures the flexural stiffness and strength of polymer matrix composites. The specimens of rectangular shape having dimensions 100 mm × 10 mm were supported at both ends and deflected at the centre. As bending force was exerted, the specimens deflected at the centre. The dimensions of length, width and thickness for each sample were entered into the system. The stress–strain curves were recorded on the system until the maximum force reached 40%. For each type of sample, 20 measurements were carried out.

**Figure 8:**
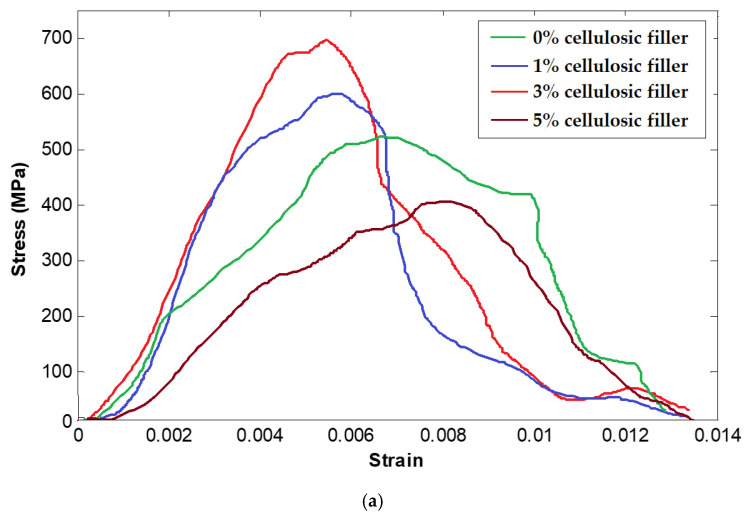

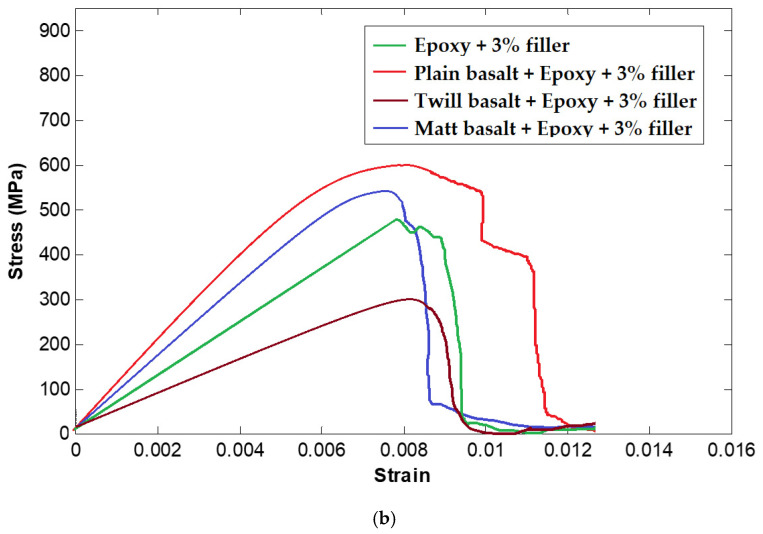
Flexural stress–strain curves for hybrid composite samples: (**a**) effect of nanocellulosic filler content, (**b**) effect of weave structure at 3% cellulosic filler.

The flexural stress–strain curves for hybrid composites partially reinforced with 1%, 3% and 5% nanocellulosic fillers are shown in Figure 8a. The charts are averaged from 20 measurements for each type of sample. Since the sample with 3% filler exhibited the maximum flexural strength, further analysis was carried out to observe the influence of basalt fabric structure on the hybrid composites reinforced with 3% nanocellulose. The influence of fabric structure on flexural performance is shown in Figure 8b.

It can be observed from Figure 8a that the flexural strength of composite samples improved after the addition of cellulose nanofillers up to 3% in the epoxy resin. However, the addition of 5% cellulose tends to decrease the flexural strength. These observations are supported by the trends obtained in the tensile strength of the samples reinforced with cellulosic fillers. The flexural modulus was also observed to increase as the hybrid composites were reinforced with cellulosic fillers up to 3% wt. The high modulus corresponds often to a lower strain to break. The interfacial bondage between nanofillers of cellulose with the molecules of epoxy resin is responsible for improved flexural performance [73,74,75]. This increased flexural modulus indicates improved toughening of epoxy with the addition of cellulose nanofillers. The decrease in strength for the 5% loading of cellulose can be attributed to the nonuniform dispersion due to the higher number of nano-sized cellulosic fillers. The formed agglomerates might have acted as stress concentration points leading to lower flexural strength and modulus. The results are supported by previous literature [76,77,78] as well as the trends of tensile strength observed in the hybrid composite samples.

The influence of basalt fabric structure on flexural performance can be observed in Figure 8b. It is well known that the plain weave is stronger as compared to other weave patterns due to intensive interlocking between the constituent yarns. It is evident from the results that plain woven basalt fabric reinforced hybrid composite partially reinforced with 3% nanocellulosic filler exhibited the best tensile and flexural performance. Among the twill and matt structures, the matt structure presents a tighter construction and better mechanical performance. Therefore, matt woven fabric-based composite performs better than twill fabric-based hybrid composites. The directional bias (inclination of twill line) of the fabric results in imbalanced force distribution and lower tensile and bending performance as demonstrated in the literature [79,80,81,82].

### 3.5. Impact Test

**Figure 9:**
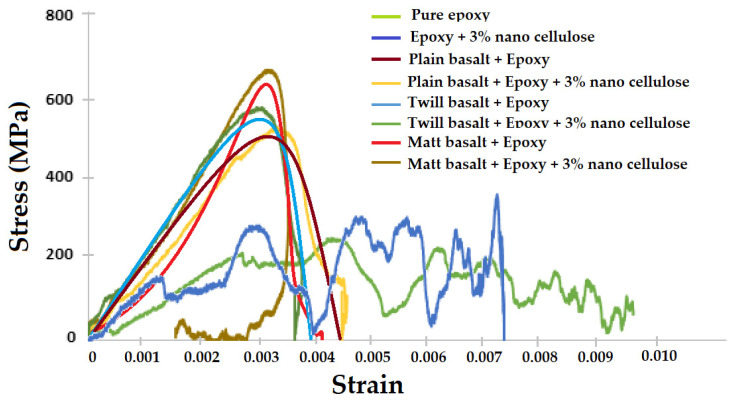
Impact stress–strain curves for developed composite samples.

Since the 3% wt of cellulosic filler proved to improve the tensile and flexural performance of the hybrid composites to the maximum extent, further evaluation of impact performance was carried out to examine the effect of fabric structure in the hybrid composites. Impact tests were carried out for 3% nanocellulose-filled hybrid composites reinforced with different basalt fabric structures. Each type of sample was tested 20 times and the mean values were calculated. The stress–strain curves of the impact test for the composite samples are shown in Figure 9. The charts are averaged from 20 measurements for each type of sample.

The impact strength (load at break) was recorded in the system and the energy at the break for various hybrid composite samples reinforced with 3% cellulosic filler was calculated as follows:

*E* = Mass of impactor (*m*) × acceleration due to gravity (*g*) × drop height (*h*) (4)

**Figure 10:**
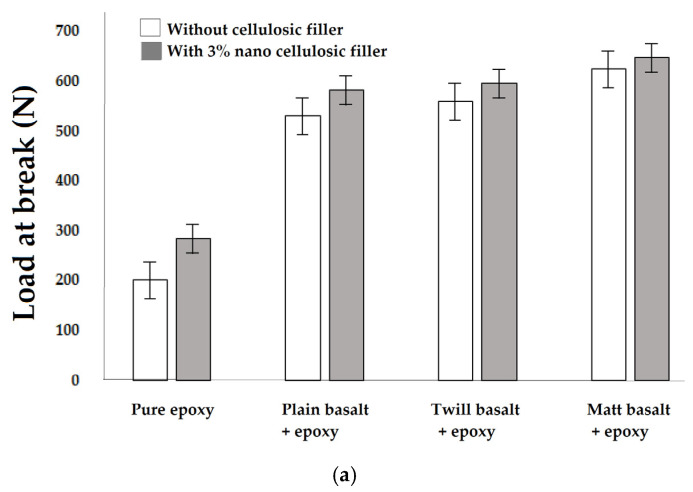

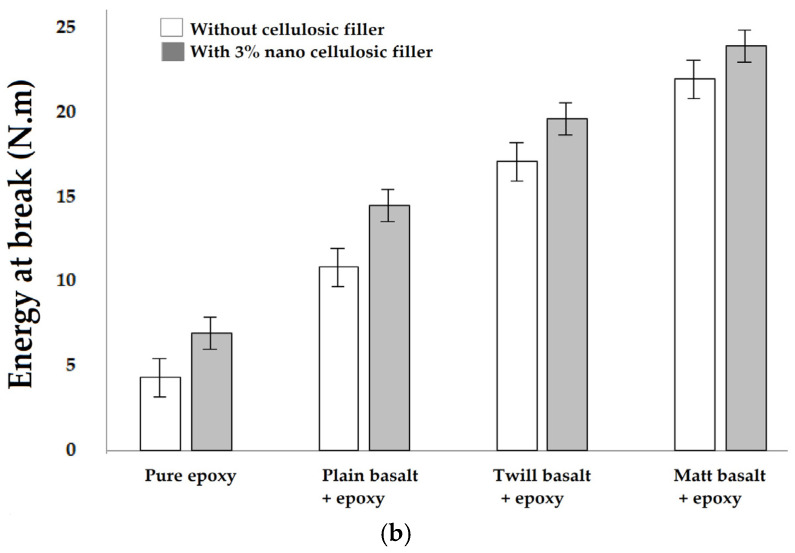
Impact performance of hybrid composite samples: effect of 3% nanocellulosic filler content on (**a**) load at break and (**b**) energy at break for different weaves.

The results are shown in Figure 10a,b, respectively. The confidence intervals are based on 20 measurements.

It was observed that the impact strength of hybrid composites improved by 6–8% upon the addition of 3% cellulosic fillers as compared with the neat fabric-based nonhybrid composites without filler. The percent increments over pure epoxy resin matrix were found to be 72.02%, 82.22% and 96.41%, corresponding to various woven constructions e.g., plain, twill and matt structures of basalt fabrics. These results suggest that the cellulosic fillers served as stress concentrators to initiate a multiplicity of small cracks around the particulates in composites, thus absorbing impact energy and diverting the subversive cracking. The branches of cracks can also help prevent the cracks from growing catastrophically. Therefore, the impact strength of composites was enhanced significantly. The reason is mechanical activation of cellulose fillers through ball milling enhanced the dispersion in epoxy with enhanced interfacial bonding between cellulose–epoxy and epoxy–basalt.

It is interesting to notice that the best impact performance (load as well as energy at break) of hybrid composites was observed with matt woven basalt fabrics followed by twill woven basalt. The worst impact performance was observed in the case of plain-woven basalt fabric-reinforced hybrid composite. These findings are in sharp contrast to the trends observed in tensile and bending performance. Such tendencies are also reported in the previous literature [83,84].

Impact performance is a multiaxial loading situation, unlike the tensile or bending deformations which are mostly uniaxial. When the impactor/ball strikes the samples at a reasonably high momentum and kinetic energy, the force is radially distributed while simultaneously being transmitted through the thickness direction [85]. In the case of plain-woven basalt fabrics, the constituent yarns and fibres are interlaced to the maximum extent and, thus, each individual layer is very firmly integrated into the axial direction. However, owing to the very intensive change of plane for the interlacing yarns, the mechanical performance in the thickness direction deteriorated. Further, the interlinking between different layers is relatively weak, which leads to delamination in certain instances. Such observations are also reported in the literature [86,87]. On the other hand, in the case of weaves involving longer floats, e.g., twill, matt or satin, the yarns present significantly longer float lengths to bond with the adjacent laminates. This enhances the mechanical performance in the thickness direction substantially. That is why matt and twill basalt-based composite samples showed better impact performance as compared to plain-woven fabric-based samples. The twill basalt structure shows a directional bias owing to the inclination of the twill line (Z or S) direction. Therefore, it is not as symmetrical as a matt weave. Ultimately in the case of the matt construction, there is a combined advantage of longer float length as well as the symmetry of the fabric structure [88].

### 3.6. Dynamic Mechanical Analysis (DMA)

**Figure 11:**
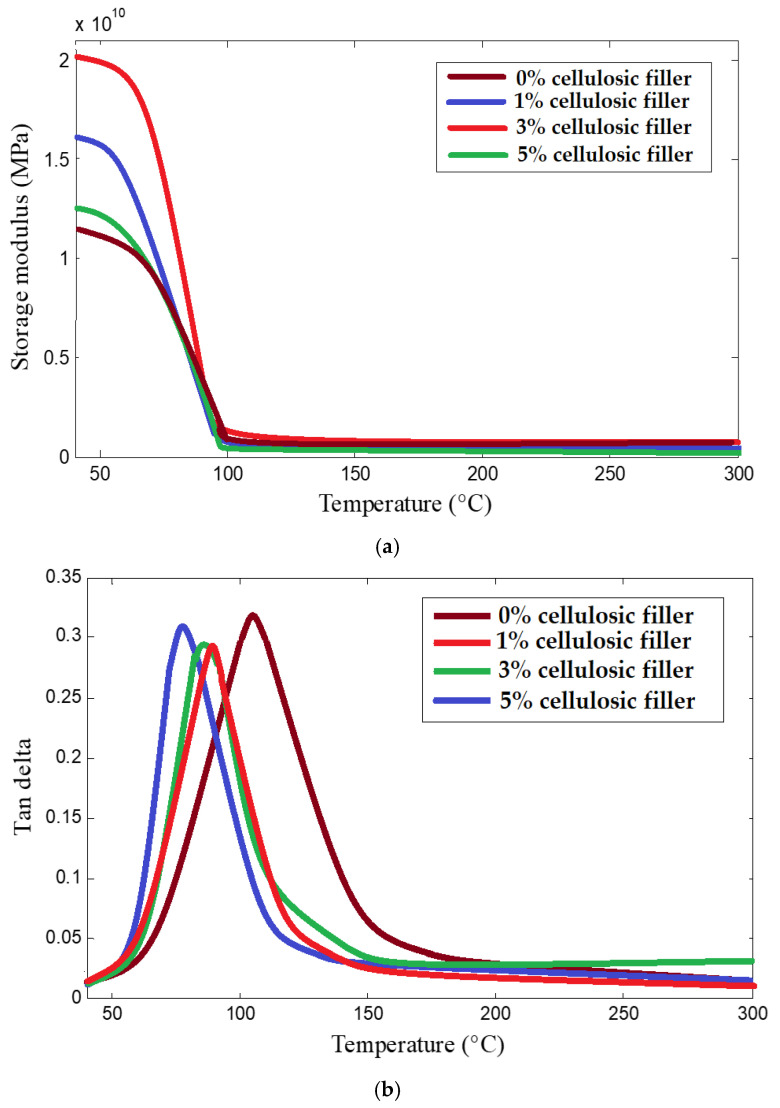
Temperature dependence of (**a**) storage modulus and (**b**) tan δ based on cellulosic filler content in the composites.

Figure 11a,b show the temperature dependence of storage modulus and tan δ, respectively, for the composites reinforced with different percentages of wet-milled cellulosic fillers. The charts are averaged from 5 measurements for each type of sample.

The storage modulus decreased sharply in the temperature range of 60 °C to 85 °C. After 100 °C, there is a plato on the curves. It was observed that the storage modulus increases as the cellulosic filler content increases from 1% to 3%. Such behaviour is attributed to the higher thermal stability of cellulosic nanofillers as compared to epoxy resin. However, for 5% cellulose content, the storage modulus decreases sharply. This could be due to the nonuniform dispersion of nanofillers at higher concentrations as was observed while analysing the mechanical performance.

The tan δ values shown in Figure 11b increase initially and then decrease with increasing temperature. It can be observed that the addition of cellulosic fillers reduced the loss modulus and consequently the tan δ of the composites. The inclusion of cellulosic fillers might have given rise to some weaker interfacial bonds as compared to a stronger and more uniform intermolecular force in the pure resin. Thus, the molecules in a cellulose-filled resin may start to destabilize at a relatively lower temperature as compared to the pure resin. This could be a reason for the lower T_g_ value for a composite with cellulosic filler as compared to pure resin.

**Figure 12:**
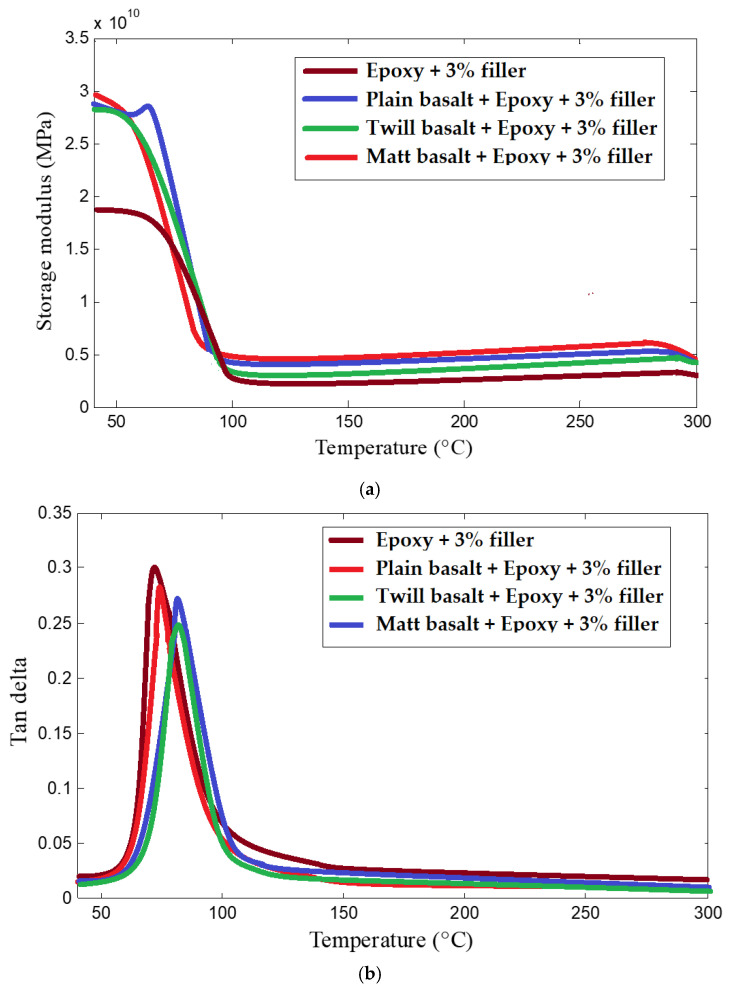
Temperature dependence of (**a**) storage modulus and (**b**) tan δ based on different basalt fabric weaves and 3% cellulosic filler content in the hybrid composites.

Figure 12a,b show the temperature dependence of storage modulus and tan δ, respectively, for the hybrid composites reinforced with different structures of basalt fabric and partially reinforced with 3% cellulosic fillers. The charts are averaged from 5 measurements for each type of sample.

It can be observed from Figure 12a that the storage modulus of hybrid composite samples increased significantly due to the basalt fabrics used as reinforcement. It is well known that basalt is highly stable at higher temperatures and, thus, it enhances the stability of the hybrid composites. Since the dynamic mechanical properties are mostly related to the microscale structure and molecular stability, the macroscale weave structures seem to have an insignificant influence on the storage modulus and tan δ. Therefore, no significant difference in thermomechanical behaviour was observed between hybrid composites using plain, twill and matt structures of the basalt fabrics.

The curves of the temperature dependence of tan δ are shown in Figure 12b. The composite samples show a peak at about 85–90 °C which is closely related to the glass transition temperature (T_g_) of the matrix (epoxy + nanocellulose). A further lowering in the peaks was observed after reinforcing the composites with basalt fabric. In other words, basalt fibres help in lowering the loss modulus and tan δ of epoxy resin along with the addition of 3% nanocellulosic fillers.

In general, the tan δ values of composites filled with cellulosic fillers are lower than that of the unfilled epoxy matrix, which proves that the addition of cellulose enhances the damping capacity of epoxy resin. The use of the basalt fabric constructions as reinforcement showed improvement in the damping capacity of hybrid composites along with the addition of cellulosic fillers. This fact may be explained by the addition of cellulose which increases the contributions of the hollow structure in cellulose and frictional damping, thereby leading to a decrease in the loss of energy, and thus decreasing the tan δ values.

Overall, both the basalt fabric reinforcement and cellulosic fillers up to 3% wt, enhance the mechanical as well as thermomechanical performance of hybrid composites considerably.

## 4. Conclusions

The hybridisation of cellulosic filler material in an epoxy matrix along with basalt woven fabric reinforcement was proved as a successful approach. The cellulosic nanofillers with a size of 450 nm were obtained after prolonger milling action. The mechanical properties of the basalt–epoxy–cellulose hybrid composites were determined and compared with those of regular basalt–epoxy composites. The tensile properties were determined numerically by using computational tools. The prediction of tensile performance showed relatively good agreement with the experimental results. At higher concentrations of the cellulosic fillers (especially beyond 3% wt), the properties cannot be predicted accurately due to experimental shortcomings relating to the nonuniform dispersion of particles. The mechanical analysis revealed that hybrid basalt–epoxy–cellulose composites perform better in terms of energy absorption compared to nonhybrid composites. The results are in agreement with previous studies dealing with nanofillers in composites [60,61,62]. The tensile and bending performance of hybrid composites proved to be optimum at 3% filler content. Among the various weaves of basalt fabric, the plain-woven structure showed the best tensile and bending performance. However, the best impact performance was observed for the matt woven basalt fabric reinforced composites. Similar results were reported by several other researchers [28,30].

Thermomechanical performance of hybrid composites improved by the addition of nanofillers up to 3% wt. The storage modulus increased while the tan δ values decreased with the addition of cellulosic fillers in the hybrid composites [33,83]. The reinforcement of basalt fabric enabled a significant increase in storage modulus irrespective of the weave type. The high-temperature resistance of basalt [16,17,19], as well as cellulosic fillers, is a contributing factor towards the enhanced thermomechanical performance of such hybrid composites. The developed samples are suitable for high-performance construction materials in housing, sports and transportation applications. For these specific applications, the material properties of basalt–epoxy–cellulose hybrid composites are comparable with widely used glass and carbon fibre composites. On the other hand, the cost of hybrid composites can be lower due to the use of inexpensive bio-based filler materials. The inclusion of biological fillers in hybrid composites is a futuristic step towards developing eco-friendly construction materials without compromising mechanical and thermomechanical performance.

## Figures and Tables

**Table 1:** Properties of fibres and yarns used.

Properties	Basalt
Diameter of fibres (micron)	12
No. of filaments	890
Linear density of yarn (Tex)	296
TPM (Twists/meter)	20 ± 1
Tensile strength (N)	92.75 ± 3.54
Tensile elongation (%)	1.29 ± 0.03
Tenacity (N/tex)	0.315 ± 0.01
Initial modulus (MPa)	9378 ± 32

**Table 2:** Properties of green epoxy resin.

Properties	Value
Physical State	Liquid at 20 °C
Colour	Yellowish to Yellow
Boiling Point	270–280 °C (at very low pressure)
Density (g/cm^3^)	1.16 at 20 °C
Water Solubility (g/L)	6–9 at 20 °C
Viscosity (Poise)	8–10 at 25 °C
Solubility	Soluble in Acetone
Storage Temperature	5–25 °C
Epoxide Index (mol/kg)	5.4–5.7
Mass Equivalent of Epoxide, EEW (g/mol)	176–186
Colour (Green)	Max. 100
Hydrolyzable Chlorine Content (%)	Max. 0.03
Non-volatile Substances (2 h/140 °C)	Min. 99.5%

**Table 3:** Details of samples.

Sample No.	Sample Code	Description	Fabric wt%	Epoxy wt%	Cellulose wt%
1	ER	Pure epoxy resin	0	100	0
2	BF + ER	Basalt fabric + epoxy resin	50	50	-
3	BF + ER + 1%MC	Basalt fabric + epoxy resin + 1% microcellulose	50	49	1
4	BF + ER + 3%MC	Basalt fabric + epoxy resin + 3% microcellulose	50	47	3
5	BF + ER + 5%MC	Basalt fabric + epoxy resin + 5% microcellulose	50	45	5
6	BF + ER + 10%MC	Basalt fabric + epoxy resin + 10% microcellulose	50	40	10
7	BF + ER + 1%NC	Basalt fabric + epoxy resin + 1% nanocellulose	50	49	1
8	BF + ER + 3%NC	Basalt fabric + epoxy resin + 3% nanocellulose	50	47	3
9	BF + ER + 5%NC	Basalt fabric + epoxy resin + 5% nanocellulose	50	45	5
10	BF + ER + 10%NC	Basalt fabric + epoxy resin + 10% nanocellulose	50	40	10

## Data Availability

Not applicable.
